# Temperature-dependent sRNA transcriptome of the Lyme disease spirochete

**DOI:** 10.1186/s12864-016-3398-3

**Published:** 2017-01-05

**Authors:** Niko Popitsch, Ivana Bilusic, Philipp Rescheneder, Renée Schroeder, Meghan Lybecker

**Affiliations:** 1 Wellcome Trust Centre for Human Genetics, University of Oxford, Oxford, UK; 2 Department of Biochemistry and Cell Biology, Max F. Perutz Laboratories, University of Vienna, Vienna, Austria; 3 Center for Integrative Bioinformatics Vienna, Max F Perutz Laboratories, Medical University of Vienna, University of Vienna, Vienna, Austria; 4 Department of Biology, University of Colorado Colorado Springs, Colorado Springs, CO USA

**Keywords:** *Borrelia burgdorferi*, sRNA transcriptome, Regulatory RNA, Antisense RNA (asRNA), Intragenic RNA (intraRNA), Riboswitch

## Abstract

**Background:**

Transmission of *Borrelia burgdorferi* from its tick vector to a vertebrate host requires extensive reprogramming of gene expression. Small regulatory RNAs (sRNA) have emerged in the last decade as important regulators of bacterial gene expression. Despite the widespread observation of sRNA-mediated gene regulation, only one sRNA has been characterized in the Lyme disease spirochete *B. burgdorferi*. We employed an sRNA-specific deep-sequencing approach to identify the small RNA transcriptome of *B. burgdorferi* at both 23 °C and 37 °C, which mimics in vitro the transmission from the tick vector to the mammalian host.

**Results:**

We identified over 1000 sRNAs in *B. burgdorferi* revealing large amounts of antisense and intragenic sRNAs, as well as characteristic intergenic and 5′ UTR-associated sRNAs. A large fraction of the novel sRNAs (43%) are temperature-dependent and differentially expressed at the two temperatures, suggesting a role in gene regulation for adaptation during transmission. In addition, many genes important for maintenance of *Borrelia* during its enzootic cycle are associated with antisense RNAs or 5′ UTR sRNAs. RNA-seq data were validated for twenty-two of the sRNAs via Northern blot analyses.

**Conclusions:**

Our study demonstrates that sRNAs are abundant and differentially expressed by environmental conditions suggesting that gene regulation via sRNAs is a common mechanism utilized in *B. burgdorferi*. In addition, the identification of antisense and intragenic sRNAs impacts the broadly used loss-of-function genetic approach used to study gene function and increases the coding potential of a small genome. To facilitate access to the analyzed RNA-seq data we have set-up a website at http://www.cibiv.at/~niko/bbdb/ that includes a UCSC browser track hub. By clicking on the respective link, researchers can interactively inspect the data in the UCSC genome browser (Kent et al., Genome Res 12:996-1006, 2002).

**Electronic supplementary material:**

The online version of this article (doi:10.1186/s12864-016-3398-3) contains supplementary material, which is available to authorized users.

## Background

Lyme disease is the most common arthropod-borne disease in the United States of America and Europe, with 300,000 cases reported annually in the US [1]. *Borrelia burgdorferi*, a causative agent of Lyme disease, oscillates in nature between a tick vector and a vertebrate host [2–4]. The enzootic life cycle of *B. burgdorferi* is maintained by uninfected tick larvae feeding on an infected vertebrate, usually a small mammal. The infected larvae molt into nymphs and the spirochetes are transmitted to and infect a vertebrate at the next blood meal [4]. The infected nymphs are also the primary route of *B. burgdorferi* transmission to humans. *B. burgdorferi* must survive in and transition between two vastly different environments, the tick vector and the vertebrate host [4, 5]. Like many other pathogenic bacteria, *B. burgdorferi* senses and responds to environmental cues, such as a change in temperature [6–9], by regulating the gene expression of proteins necessary for survival [4, 5].

Bacterial gene expression is highly regulated at the level of transcription, which is catalyzed by RNA polymerase (RNAP) and regulated by transcription factors. The RNAP sigma factor is responsible for promoter selectivity. Many bacteria synthesize several different sigma factors, with different promoter selectivity, thus directing RNAP to a discrete set of genes, which results in the control of a set of genes needed for a certain response [10]. *B. burgdorferi* has only three sigma factors (RpoD, RpoS and RpoN), a relatively small number compared to other bacteria, which can encode up to eighteen [10]. Moreover, transcription of *rpoS* is regulated by RpoN, effectively decreasing the regulatory breadth of sigma factors in *B. burgdorferi* [11]. Transcription is also regulated via several characterized transcription factors in *B. burgdorferi* [5, 12–14]*.* However, little is known about post-transcriptional gene regulation in this spirochete.

Posttranscriptional gene regulation via a variety of regulatory RNAs has emerged in the past decade as a major mechanism of modulating gene expression [15–18]. The most extensively studied regulatory RNAs in bacteria are the *trans*-encoded small RNAs (sRNAs) that are predominately encoded in intergenic regions between two annotated genes. Most *trans*-encoded sRNAs regulate gene expression by imperfectly base pairing with a target mRNA and affecting the translation and/or the stability of the mRNA. The RNA chaperone Hfq is often required for sRNA:mRNA base-pairing [19]. *Cis*-encoded antisense RNAs (asRNAs), which are RNAs transcribed opposite to annotated genes, have been ubiquitously reported [20–22]. asRNAs have complete complementarity to their sense mRNA counterpart and were originally identified opposite to phage, toxin and transposon genes [23, 24]. asRNAs act in a manner similar to *trans*-encoded sRNAs via binding their cognate mRNA and influencing its translation and/or stability [20, 21]. However, asRNAs can also regulate their sense mRNAs transcription via transcriptional interference [20, 21, 24, 25]. Riboswitches are another *cis*-acting class of regulatory RNAs encoded in long 5′ UTRs of the mRNAs they regulate. Riboswitches usually function as sensors of metabolic cues or temperature changes [26–28]. The structure of the riboswitch is affected either by binding of a metabolite or changes in temperature stimulating or inhibiting transcription or translation of the gene. Non-coding RNAs can also act as regulators by binding proteins and sequestering them or affecting their activity [29, 30]. Furthermore, intragenic sRNAs are a new class of sRNAs that are encoded within annotated genes [31]; relatively little is known about their function. Finally, dual-function RNAs encode both a regulatory RNA and a protein. Staphylococcal RNAIII regulates the translation of several genes via imperfect base pairing and encodes a small (25 amino acid) hemolysin peptide [32, 33].

sRNAs are recognized as important regulators of many adaptive and physiological gene expression changes in pathogenic bacteria [34–36]. However, despite the pervasive nature of regulatory RNAs, only one regulatory RNA has been identified and characterized to date in *B. burgdorferi* [37]. *B. burgdorferi* encodes two characterized RNA-binding proteins, a unique Hfq protein (Hfq_Bb_) (39), which is required for murine infection via needle inoculation, and a homolog of CsrA (CsrA_Bb_), although there is controversy regarding its function in infection of the mammalian host [38–41]. CsrA normally acts in a concerted manner with two non-coding RNAs, which have not been identified in *B. burgdorferi*. We hypothesized that *B. burgdorferi* has a large sRNA network that is required for transducing the enzootic life cycle and pathogenesis. Here we specifically identified the sRNA transcriptome of *B. burgdorferi* at 23 °C and 37 °C, temperatures that mimic the tick vector and vertebrate host, respectively, and we found a large sRNA network. This study is the first transcriptome-wide analysis of sRNAs in the Lyme disease spirochete.

## Results/discussion

### Transcriptome-wide identification of small RNAs

The main goal of this study was to identify small RNAs in *B. burgdorferi* that are important for gene regulation associated with the enzootic cycle of the spirochete [4, 5]. Temperature is one of the key environmental stimuli that modulate gene expression in *B. burgdorferi* [6–8]. For this reason, we shifted the temperature of *B. burgdorferi* growing in liquid culture from 23 °C to 37 °C to mimic transmission from the tick vector to the vertebrate host and deep-sequenced the small RNA transcriptome from these cultures.

Stranded cDNA libraries were prepared from the ribosomal RNA-depleted size-selected (50–500 nt) RNA. Three independent biological replicates were sequenced. To obtain the most complete coverage of the *B. burgdorferi* genome and capture also lowly expressed sRNAs, the first biological replicates (23 °C and 37 °C) were each sequenced on a single lane of an Illumina HiSeq 2000 and mapped to the *B. burgdorferi* strain B31 reference genome (replicate 0, rep0). This resulted in 170 and 190 million mapped 50 bp single-end (SE) reads, respectively, which corresponds to very deep theoretical genomic coverages of about 5600X and 6200X. Two additional replicates (rep1, rep2) were sequenced to lower coverage (27–44 million mapped reads, 900-1400X, see Table 1) and were used for validation and differential gene expression analysis.

**Table 1:** Sequencing and mapping statistics

	Temp	# Raw reads	# Mapped reads	%	Theoretical coverage	*Used for*
*rep0*	23 °C	238,560,232	191,196,698	80%	6284 X	*sRNA search*
	37 °C	220,892,146	171,375,012	78%	5633 X	
*rep1*	23 °C	48,549,216	36,639,898	75%	1204 X	*Differential expression analysis*
	37 °C	44,318,061	31,718,243	72%	1043 X	
*rep2*	23 °C	54,118,451	44,988,440	83%	1479 X	
	37 °C	46,386,965	27,538,869	59%	905 X	

All data was sequenced on an Illumina Hiseq2000. Reads are 50 bp single-end

For the accurate identification of *B. burgdorferi* sRNAs, we extracted strand-specific coverage signals from our deep sequencing datasets and developed a simple computational method to search these data for sRNAs-derived coverage peaks (see Methods). This method identified peaks stemming potentially from sRNAs in both deep data sets (23 °C and 37 °C) that were then merged in order to get a final set of genomic candidate intervals (sRNAs) based on the peak borders, see Methods. This strategy and the depth of coverage of our data sets allowed us to identify sRNAs expressed at low levels as well as sRNAs expressed in a temperature-dependent fashion. We acknowledge, however, that our method may have missed some very lowly expressed sRNAs due to the thresholds we configured for reducing the number of false positive calls (e.g., a minimum peak height of 500 reads), see Methods. As proof of principle, our method identified known and annotated tRNAs, RNase P, tmRNA, 4.5S RNA, 6S RNA (S. Samuels personal communication) and DsrA_Bb._ For example, coverage maps illustrate the peaks called for two phenylalanine tRNAs (Additional file 1: Figure S1).

**Fig. 1:**
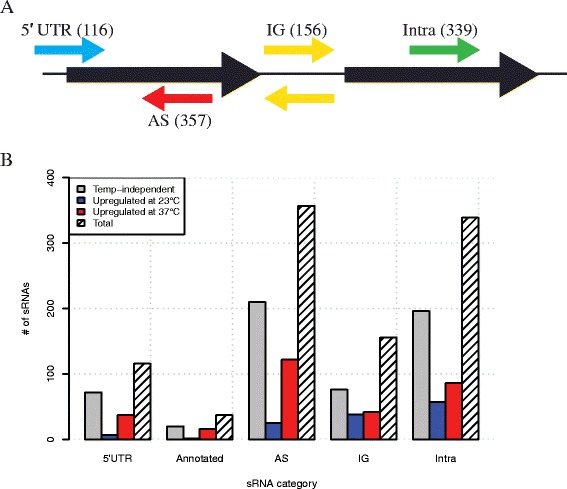
Genomic location, categorization and temperature-dependence of small RNAs. **a** sRNAs were categorized according to their genomic context as 5′ UTR sRNAs (*blue arrow*), antisense (AS) RNAs (*red arrow*), intergenic (IG) RNAs (*yellow arrows*), or intragenic (intra) RNAs (*green arrow*). Annotated coding features (ORFs) are represented as black arrows. The numbers of sRNAs identified in each category are in parenthesis next to the category. **b** A bar chart illustrates the numbers of temperature-dependent and -independent sRNAs identified in each category. Gray bars are temperature-independent sRNAs, blue bars are sRNAs up-regulated at 23 °C compared to 37 °C and red bars are sRNAs up-regulated at 37 °C compared to 23 °C

The resulting list of 5,600 genomic intervals identified by our peak caller were then manually curated by multiple members of our research group by visual inspection of the normalized coverage data in the IGV genome browser [42] resulting in 1,005 putative sRNAs (Additional file 2: Table S1) that were classified based on their genomic context (Fig. 1a). 116 of the called genomic intervals overlapped the 5′ end of an annotated open reading frame and were classified as a 5′ UTR sRNAs. 156 of sRNAs were transcribed between annotated open reading frames (ORFs) and were classified at intergenic sRNAs (IG-sRNAs). 357 sRNAs were found to be transcribed from the opposite strand to an annotated open reading frame and were classified as *cis*-encoded antisense RNAs (asRNA). 339 sRNAs were identified within annotated ORFs and were classified as intragenic sRNA (intraRNAs). The remaining 37 sRNAs were previously known and annotated accordingly (tRNAs, 5S RNAs, ffx (4.5S RNA), SsrA, DsrA and RNase P). For the categorization of the sRNAs, we took a simplified approach (the as-, intra- and 5′ UTR RNAs only needed one base of the interval to overlap with the open reading frame) and acknowledge that, depending on the genomic context, intraRNAs, IG RNAs and asRNAs may be 5′ UTRs for proximal genes; however, this cannot be determined from our sRNA sequencing. In summary, our search strategy successfully detected all 37 previously annotated sRNAs and additionally identified 968 novel sRNAs. In addition, an *in silico* analysis of the nucleotide context surrounding the detected 5′ ends of the sRNAs identified an adenine and thymine rich −10 region correlating to the Pribnow box providing additional evidence for the accuracy of our approach (Additional file 3: Figure S7).

RNA-seq results including normalized coverage tracks and all identified sRNAs are available at http://www.cibiv.at/~niko/bbdb/. The data can be downloaded or directly viewed in the UCSC Genome Browser [43]. Note that the normalization method used to generate the coverage tracks for visualization differs from the method used by differential gene expression analysis programs (EdgeR and DESeq). Therefore, visual inspection of sRNA coverage signals cannot be used to determine statistically significant gene expression changes.

### Temperature-dependent sRNAs

We identified the sRNA transcriptome at both 23 °C and 37 °C to elucidate sRNAs involved in temperature-dependent gene regulation associated with the enzootic cycle. Stranded sRNA transcriptomes from the two biological low-coverage replicates at both temperatures were sequenced and annotated sRNAs from our peak-finding algorithm were used to test for statistically significant differential expression at the two temperatures using EdgeR. This method identified 431 (43%) of the sRNAs as temperature-dependent and differentially regulated at the two temperatures: 128 sRNAs were up-regulated at 23 °C, while 303 were up-regulated at 37 °C. 22 sRNAs were validated by Northern blot analyses (Additional file 4: Table S2 and Additional file 5: Figure S2, Additional file 6: Figure S3, Additional file 7: Figure S4, Additional file 8: Figure S5 and Additional file 9: Figure S6) and temperature-dependent and temperature-independent sRNAs were identified in all categories of sRNAs (Fig. 1b).

### *Cis*-encoded small RNAs

**Fig. 2:**
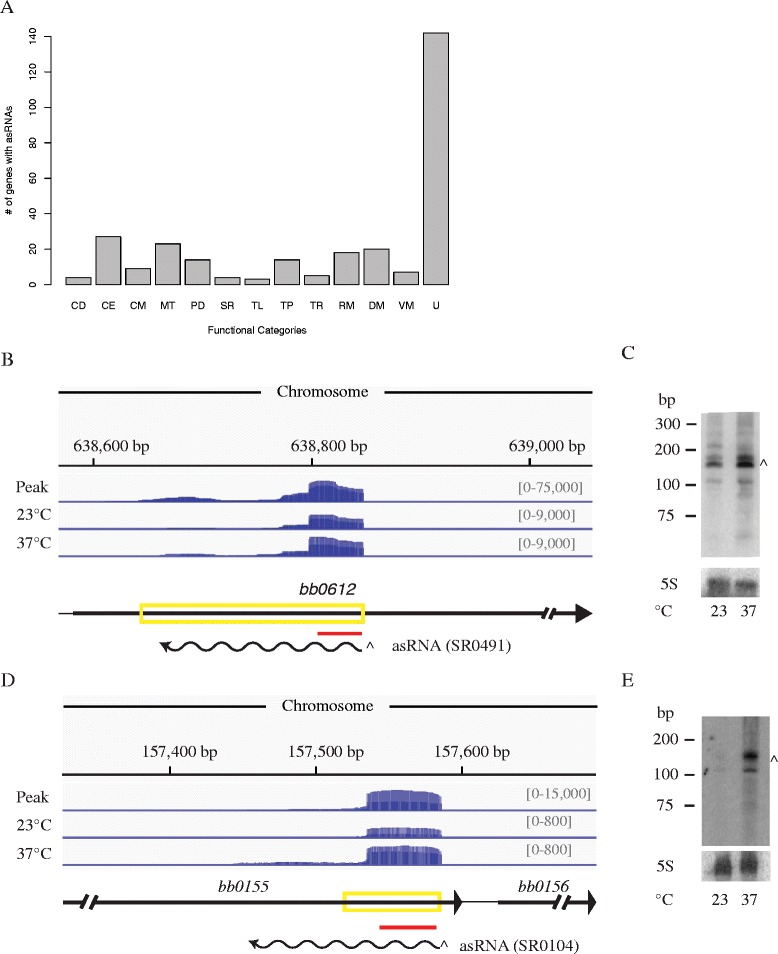
Identification and confirmation of small asRNAs. **a** Genes with asRNAs encoded opposite to them are functionally categorized using the following abbreviations: CD, cell division; CE, cell envelope; CM, cell motility; MT, metabolism; PD, protein degradation and folding; SR, stress response; TL, translation; TP, transporter; TR, transcription; RM; RNA metabolism; DM, DNA metabolism; VM, vitamin metabolism and U, unknown. asRNAs (SR0493 and SR0104) identified by sRNA-seq were confirmed via Northern blot analyses for as-*bb0155* and as-*bb0612*. **b** and **d** The deep sequencing results are displayed in a coverage map of an overlay of the two rep0 libraries sequenced deeply for sRNA peak calling (Peak) and the overlay of the two biological replicates rep1, rep2 at both 23 °C and 37 °C. The height at each position indicates the normalized number of reads that mapped to that base. The – strand coverage is shown in blue. Note that the *y*-axis scale is different between the peak calling libraries (peak) and the biological replicates used for differential expression analyses (indicated by the grey numbers in brackets). The genomic context is illustrated below the coverage maps: black arrows indicate the annotated ORFs, the yellow box indicates the region called as a small asRNA by our peak finder and the wavy line is the putative transcript determined by Northern blot. The red line represents the location of the oligonucleotide probes used for the Northern blot. **c** and **e** Northern blot analyses of total RNA fractionated on a denaturing polyacrylamide gel, blotted to a nylon membrane, and hybridized with oligonucleotides. The predicted transcripts are denoted and marked with the appropriate band (^ for sRNA) in the Northern blot. Two independent experiments were performed and representative data are shown

asRNAs are transcribed opposite to annotated ORFs and have some portion completely complementary to the corresponding sense mRNA. asRNAs vary in size from a few hundred nucleotides to 6.5 kb [20, 21]. We identified 357 small asRNAs: 147 are temperature-dependent; 25 are up-regulated at 23 °C, while 122 are up-regulated at 37 °C (Fig. 1b). Overall, 296 out of 1569 annotated genes have at least one asRNAs encoded opposite to them and 53 of these have more than one associated asRNA (Additional file 10: Table S3). asRNA-dependent gene regulation occurs via base pairing with its cognate sense mRNA and inhibiting or stimulating expression via different mechanisms. Predominately, genes with asRNAs associated with them are hypothetical ORFs with unknown functions (Fig. 2a). Notably, asRNAs were identified opposite to key genes active in the maintenance of the *B. burgdorferi* life cycle and cell division, such as: *bb0420* (*hk1*), *bb0827* (*hrpA*), *bbb03* (*resT*), *bbb17* (*guaB*), and *bba66* [4, 13, 44–50]. Hk1 is the histidine kinase in the two-component system necessary for *B. burgdorferi* survival in the tick [45]. There are two as-*hk1* RNAs, which are both up-regulated at 37 °C. The *hk1* transcript is expressed at low levels in vivo in both the flat tick, fed tick and in *B. burgdorferi* grown in dialysis membrane chambers (mimicking the mammalian host). However, the levels were the highest in larvae fed to repletion and flat ticks [45]. We hypothesize that the as-*hk1* RNAs play a role in the fine-tuning of gene regulation of *hk1*. There are also two as-*resT* RNAs, one is temperature independent and the other is up-regulated at 23 °C. ResT is the telomere resolvase required for telomere resolution during replication of the linear and circular genetic elements [51]. Finally, there are two asRNAs, both up-regulated at 37 °C, encoded opposite to *bba66*, which is required for mouse infection via tick transmission [44].

In 2002, microarray analysis identified 79 genes up-regulated and 15 genes down-regulated at 37 °C compared to 23 °C [52]; 17 of these 94 genes have asRNAs associated with them. Eight of these asRNAs are temperature-dependent in our data; two of the sense/as RNA pairs are reciprocally regulated, while six are co-regulated (Additional file 11: Table S4). These data hint at a molecular mechanism for gene regulation by the asRNAs. For example, *bb0385* is down-regulated at 37 °C compared to 23 °C in the microarray data [52], while the as-*bb0385* RNA (as-*0385*) is up-regulated at 37 °C compared to 23 °C in our RNA-seq data. We hypothesize that the asRNA, when up-regulated at 37 °C, binds to the mRNA and inhibits transcription or initiates degradation effectively lowering the levels of the mRNA. For the mRNAs and asRNAs that are up or down-regulated together, we propose that the asRNA stabilizes the mRNA and/or stimulates transcription of the gene, or acts *in trans* on a different mRNA.

RNA-seq data were validated by Northern blot analyses of five of the asRNAs (Additional file 4: Table S2 and Additional file 5: Figure S2). We identified asRNAs encoded opposite to all regions of their corresponding mRNAs; for example, the *bb0612* (*clpX*) gene has an asRNA transcribed opposite to its 5′ end and the hypothetical open reading frame *bb0155* has an antisense RNA encoded opposite to it’s 3′ end. Northern blot analyses validate the RNA-seq data and demonstrate that both as-*0155* and as-*0612* RNA steady-state levels are higher at 37 °C compared to 23 °C (Fig. 2b–d).

**Fig. 3:**
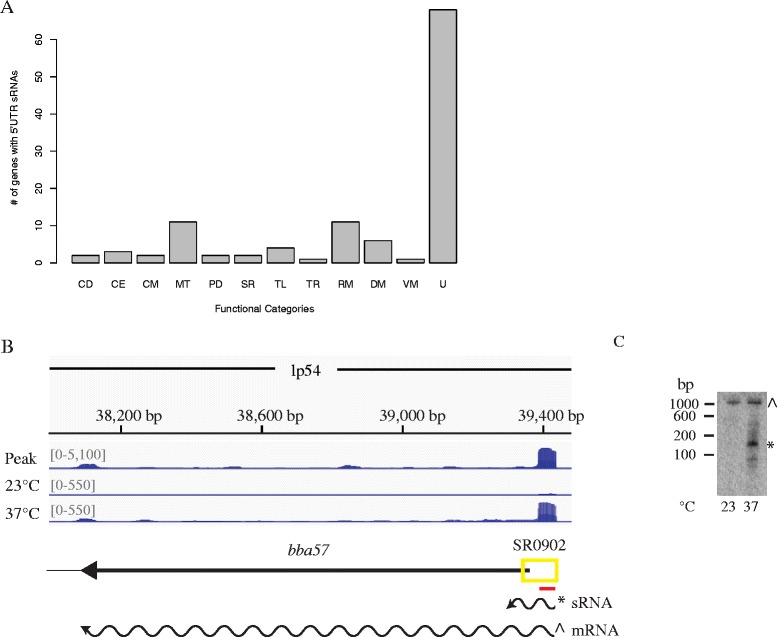
Identification and verification of 5′ UTR small RNAs. **a** Genes with small RNAs encoded in their 5′ UTRs are functionally categorized using the following abbreviations: CD, cell division; CE, cell envelope; CM, cell motility; MT, metabolism; PD, protein degradation and folding; SR, stress response; TL, translation; TR, transcription; RM; RNA metabolism; DM, DNA metabolism; VM, vitamin metabolism and U, unknown. **b** A 5′ UTR small RNA (SR0902) was confirmed via Northern blot analyses for *bba57.* The deep sequencing results are displayed in a coverage map as described in the Fig. 2 legend, except the yellow box indicates the region called as a small 5′ UTR RNA by our peak finder. **c** Northern blot analyses of total RNA fractionated on a denaturing polyacrylamide gel, blotted to a nylon membrane, and hybridized with oligonucleotides. The predicted transcripts are denoted and marked with the appropriate band (* for sRNA and ^ for mRNA) in the Northern blot. Two independent experiments were performed and representative data are shown

Stable small RNAs originating from 5′ and 3′ UTRs have been reported in several different pathogenic bacteria [18, 31, 53–59]. *Cis*-acting transcriptional riboswitches in 5′ UTRs terminate transcription in the absence or presence of metabolites and regulate gene expression of the associated mRNA. Early termination of transcription results in small RNA byproducts, which can act as *trans*-encoded sRNAs regulating gene expression of another mRNA. We identified 116 sRNAs associated with the 5′ UTR’s of annotated ORFs, suggesting they may act as riboswitches and/or *trans*-encoded sRNAs. Riboswitches often control expression of genes for transport or synthesis of key metabolic compounds and genes involved in physiological changes, virulence and stress responses. The majority of the genes with 5′ UTR sRNAs associated with them are hypothetical ORFs of unknown function and genes associated with metabolism and RNA and DNA metabolism (Fig. 3a). Of note, the transcriptional regulator BosR [14] has a 5′ UTR up-regulated at 37 °C. 44 of the 5′ UTR sRNAs were differentially regulated by temperature, suggesting these RNA elements may act as a type of transcriptional thermosensor, riboswitches that respond to temperature changes, rather than a ligand. To validate the RNA-sequencing data, we performed Northern blot analyses on two of the 5′ UTR sRNAs (Additional file 2: Table S1 and Additional file 6: Figure S3) Northern blot analysis of the 5′ UTR sRNA associated with *bba57* confirms the presence of an sRNA at 37 °C and mRNA at both 23 °C and 37 °C (Fig. 3b and c). We hypothesize that this sRNA may act *in-trans* to regulate a different mRNA or could be a transcriptional RNA thermometer. RNA thermometers are highly structured RNAs in the 5′ UTR of mRNAs that usually mask the ribosome-binding site at low temperatures inhibiting translation. At higher temperatures the structure melts and the ribosome-binding site is accessible allowing for efficient translation [60, 61]. To our knowledge no transcriptional RNA thermometers have been described. We strictly categorized 5′ UTR sRNAs, requiring the intervals we call in this category to overlap with the 5′ end of an annotated ORF. However, some of the intergenic, intragenic or antisense sRNAs may be associated with the 5′ UTRs of downstream genes and still function as riboswitches.

### Intragenic small RNAs

**Fig. 4:**
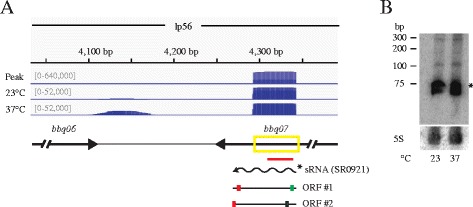
Identification and confirmation of small intragenic RNAs. The intraRNA (SR0921) was confirmed via Northern blot analyses for intra-*bbq07*. **a** The deep sequencing results are displayed in a coverage map as described in Fig. 2, except the yellow box indicates the region called as a small intraRNA by our peak finder. Putative ORFs are indicated by black lines with the canonical start codon (AUG) illustrated by a green box, the non-canonical start codon illustrated by the green box with a black outline, and stop codons indicated by a red box. **b** Northern blot analyses of total RNA fractionated on a denaturing polyacrylamide gel, blotted to a nylon membrane, and hybridized with oligonucleotides. The predicted transcripts are denoted and marked with the appropriate band (* for sRNA) in the Northern blot. Two independent experiments were performed and representative data are shown

Intragenic small RNAs (intraRNAs) are a relatively new class of sRNAs encoded from within protein coding regions. We identified 339 intraRNAs encoded within 287 annotated genes. 243 genes have one intraRNA encoded within them, while 44 genes have more than intraRNA associated with them. Intragenic RNAs have been identified via genome-wide transcriptional start site identification or co-immunoprecipitations with Hfq in several different bacteria. The genome size of *Helicobacter pylori* is similar to *B. burgdorferi* and 439 internal transcriptional start sites were identified globally. Our data identified 143 temperature-dependent intraRNAs: 86 were up-regulated at 37 °C, while 57 were up-regulated at 23 °C (Fig. 1b). Several genes important for maintenance of *B. burgdorferi* in its enzootic cycle also encode intraRNAs including [4]: *bb0382* (*bmpA*), *bb0365*, *bb0419* and *bb0420* (*rrp1* and *hk1,* respectively), *bb603*, *bbb18* (*guaA*), *bbk17*, *bbk32*, *bba16* (*ospB*), *bba64,* and *bba66*. For instance, we identified a temperature-independent intraRNA encoded at the 3′ end of the hypothetical open reading frame *bbq07* and confirmed it via a Northern blot (Fig. 4). *bbq07* potentially encodes two small ORFs; one is a truncated version of the *bbq07* ORF with a canonical AUG start codon, while the other would utilize an alternative start codon UUG and encode a different ORF. Both putative proteins would be small peptides, 27 and 33 amino acids, respectively. Relatively little is known about the function of intraRNAs and they could encode non-coding regulatory RNAs or mRNAs for small peptides. Most intraRNAs have been identified through genome-wide transcriptional start site analyses or co-immunoprecipitation with Hfq and few have been functionally characterized [31, 55, 59, 62]. However, the only characterized sRNA in *B. burgdorferi*, DsrA_Bb,_ is an intragenic RNA encoded from within *bb0577* that post-transcriptionally regulates the alternative sigma factor RpoS [37]. Recently, a small RNA processed from the 3′ UTR of an mRNA in *Salmonella* was shown to act *in* *trans* to regulate another mRNA [54]. The identification of intraRNAs greatly increases the coding potential of the relatively small genome of *B. burgdorferi*.

### Intergenic sRNAs

**Fig. 5:**
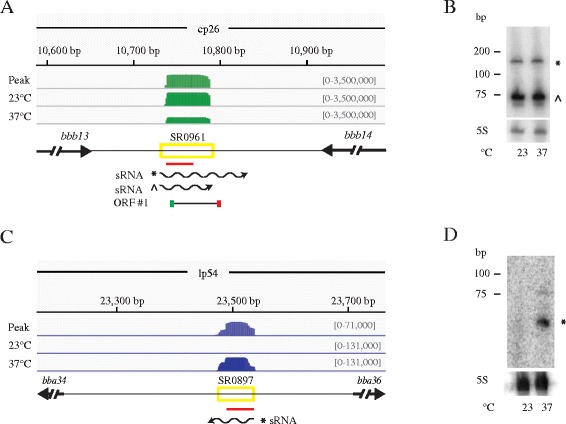
Identification and verification of intergenic small RNAs. IG**-**sRNAs (SR0961 and SR0897) were confirmed by Northern blot analyses. **a** and **c** The deep-sequencing results for the IG-sRNAs between *bbb13* and *bbb14* and between *bba34* and *bba36* are displayed in a coverage map as described in Fig. 2. A putative ORF is indicated by a black line with a canonical start codon (AUG) illustrated by a green box and the stop codon indicated by a red box. **b** and **d** Northern blot analyses of total RNA fractionated on a denaturing polyacrylamide gel, blotted to a nylon membrane, and hybridized with oligonucleotides. The predicted transcripts are denoted and marked with the appropriate band (*,^ for sRNA) in the Northern blot. Two independent experiments were performed and representative data are shown

The most well-studied class of sRNAs are encoded intergenically (IG-sRNA) and act *in trans* by base pairing imperfectly with an mRNA, encoded from another genomic region, and regulating its expression. Currently the number of IG-sRNAs is approximately 300 in both *E. coli* and *Salmonella enterica*. Moreover, in *Yersinia pseudotuberculosis* the sRNA transcriptome identified 150 novel intergenic sRNAs. Here, we identified 156 intergenic sRNAs; 42 are up-regulated at 37 °C and 38 are up-regulated at 23 °C (Fig. 1b). Considering the small genome size of *B. burgdorferi*, 156 IG-sRNAs seems on par with other well-studied bacteria. Northern blot analyses validate 13 IG-sRNAs (Additional file 4: Table S2, Additional file 8: Figure S5 and Additional file 9: Figure S6). The sRNA encoded between *bbb13* and *bbb14* is not differentially expressed at 23 °C or 37 °C in the RNA-seq data and has similar steady-state levels at both temperatures (Fig. 5a and b). In contrast, the IG-sRNA encoded between *bba34* and *bba36* is up-regulated at 37 °C in the RNA-seq data and has higher steady-state levels at 37 °C in the Northern blot analyses (Fig. 5c and d). Like other intergenically encoded sRNAs, we hypothesize that many of the IG-sRNAs regulate their target mRNAs via a variety of mechanisms requiring imperfect base pairing between the sRNA and the target mRNA. However, these sRNAs could also code for peptides. For example, the sRNA encoded between *bbb13* and *bbb14* has a putative small ORF (28 amino acids) (Fig. 5a).

### Small open reading frames

sRNAs are considered primarily non-coding and function as riboregulators. However, there are several reports of dual-function sRNAs that act as a riboregulator and code for a small peptide. The sRNA RNAIII in *Staphylococcus aureus* codes for a regulatory RNA and a 26 amino acid peptide [32, 33]. In addition, small ORFs are emerging as important components of cells in both bacteria and eukaryotes [63, 64]. We examined the coding-potential of all of the 1005 sRNAs we identified, searching for any ORFs at least 25 amino acids in length using canonical start codons. 988 of the sRNAs putatively encode at least one 25 amino acid ORF. Although this is not an accurate indicator of protein-coding sRNAs, it does demonstrate the capacity of *B. burgdorferi* sRNAs to code for small peptides.

## Conclusion

sRNAs are now universally considered to be major regulators of gene expression in pathogenic bacteria [18]. However, almost nothing was known about sRNAs in the Lyme disease spirochete, *B. burgdorferi* [37, 39]. Here we report a large temperature-dependent and -independent sRNA transcriptome in *B. burgdorferi*. The identification of massive antisense and intragenic sRNAs impacts the broadly used genetic approach to studying gene function in *B. burgdorferi*. There are now profound implications for mutagenesis experiments: deletion of a gene could include the loss of an intraRNA and/or asRNA with pleiotropic consequences. The abundance of sRNAs in the *B. burgdorferi* and their association with genes required for maintenance of its enzootic cycle suggest post-transcriptional gene regulation plays a significant role in pathogenesis.

## Methods

### Bacterial strains

Low passage *B. burgdorferi* strain B31-5A4 was grown at 23 °C and shifted to either 23 °C or 37 °C in BSK-H medium (Sigma) and grown to a low cell density 2 to 5 × 10^7^ cells/mL. Infectious *B. burgdorferi* strain B31-5A4 is a clone of the B31 strain used in Revel et al. Linear plasmid 5 was the only plasmid missing from the B31-5A4 strain used in this study.

### RNA isolation and library preparation

Total RNA was isolated using a hot phenol protocol. Total RNA was treated with DNase I (Roche) following the manufacturer’s protocol. RNA integrity was measured using the Agilent 2100 Bioanalyzer. RNA with an RNA Integrity Number (RIN) above 9.0 was used for cDNA library construction. Directional (strand-specific) RNA-seq cDNA libraries were constructed with a ligation based protocol as previously described with an initial size-selection instead of fragmentation [22, 65]. Briefly, ribosome-depleted (Ribo-Zero RNA removal kit for gram negative bacteria; Epicentre) total RNA was size fractionated on an 8% TBE-UREA gel. RNA was eluted from gel slices correlating to 50 to 500 nucleotides. RNA was treated sequentially with tobacco acid pyrophosphatase (Epicenter) and calf intestinal phosphatase (New England Biolabs) per the manufacturer’s protocols to remove 5′ tri- and monophosphates. A 3′ RNA adaptor, based on the Illumina multiplexing adaptor sequence (Oligonucleotide sequences © 2007–2014 Illumina, Inc. All rights reserved) blocked at the 3′ end with an inverted dT (5′-GAUCGGAAGA GCACACGUCU [idT]-3′), was phosphorylated at the 5′ end using T4 PNK (New England Biolabs) per the manufacturer’s protocol. The 3′ multiplex RNA adaptor was ligated to the 3′ ends of the RNA using T4 RNA ligase I (New England Biolabs). RNA was incubated at 20 °C for 6 h in 1X T4 RNA ligase reaction buffer with 1 mM ATP, 20 μM 3′ RNA adaptor, 1 μl DMSO, 5 U of T4 RNA ligase I, and 40 U of RNasin (Promega) in a 10 μl reaction. RNA was gel purified and size-selected (75–550 nt) and purified over a denaturing 8% polyacrylamide/8 M urea/TBE gel. Gel slices were incubated in RNA elution buffer (10 mM Tris–HCl, pH 7.5, 2 mM EDTA, 0.1% SDS, 0.3 M NaOAc) with vigorous shaking at 4 °C overnight. The supernatant was subsequently ethanol precipitated using glycogen as a carrier molecule. The RNAs were phosphorylated at the 5′ ends using T4 PNK (New England Biolabs) per the manufacturer’s protocol to allow for subsequent ligation of the 5′ RNA adaptor. The Illumina small RNA 5′ adaptor (5′-GUUCAGAGUU CUACAGUCCG ACGAUC-3′) was ligated to the libraries using T4 RNA ligase I (New England Biolabs). The ligated RNAs were size-selected (100–600 nt) and gel-purified over a denaturing 8% polyacrylamide/8 M urea/TBE gel (as described above). The di-tagged RNA libraries were reverse-transcribed with SuperScript®II reverse transcriptase (Invitrogen) using random nonamers per the manufacturer’s protocol. cDNA was amplified in PCR performed using Phusion® High-Fidelity Polymerase (New England Biolabs). cDNA was amplified with Illumina-compatible PCR primers by 18 cycles of PCR. The products were analyzed on an Agilent 2100 Bioanalyzer.

### Deep-seq analyses

Three independent biological replicates were sequenced on an Illumina HiSeq2000 (single-end, read length: 50 bp) to different genomic coverages (Table 1). The high-coverage replicate (rep0) was used for the identification of *B. burgdorferi* sRNAs, the two low-coverage replicates (rep1, rep2) were used to identify sRNAs that are differentially expressed at the two probed temperatures. Sequencing adapters were removed from the data using cutadapt v1.2.1 [66]. The resulting reads were mapped to the *B. burgdorferi* B31 genome (GenBank Ids: AE000783, AE001583, AE000793, AE001582, AE000785, AE000794, AE000786, AE000784, AE000789, AE000788, AE000787, AE000790, AE001584, AE000791, AE000792, AE001575, AE001576, AE001577, AE001578, AE001579, AE001580, and AE001581) using NextGenMap v 0.4.5 with default parameters and minimum identity set to 90%. Finally, multi-mapped reads were removed and strand-specific coverage data was extracted using bedtools v2.25 [67]. We developed a simple peak-calling algorithm for identifying potential sRNA peaks in these coverage signals. Briefly, the strand-specific coverage signal is read and smoothed using a moving Gaussian kernel. Then, peaks are called with a wavelet-transform based approach that uses a simple mirrored sawtooth kernel for the detection of potential peak boundaries. Peaks were called only if they complied to predefined minimum/maximum dimensions, based on our size selection (peak width: 45–500 bp, minimum peak height: 500 reads). Additionally, peaks were filtered based on their shape as we expected sRNA-derived peaks to present a sharp rise at the 5′ end and an overall “boxy” shape (i.e., a minimum height/width ratio), as seen with the positive control tRNAs (Additional file 1: Figure S1). sRNA libraries were generated from non-fragmented, size-selected RNAs and sequenced from a single end; therefore, we expected and observed a strong 5′ end bias in the sRNA coverage, which we took advantage of to determine the 5′ end of the sRNA. However, coverage of the entire sRNA is unlikely and due to capturing the degradation products of RNAs, thus making it difficult to accurately and consistently determine the 3′ end of the sRNAs. Our peak-calling algorithm, together with manual curation accurately identified intervals that correlate to sRNAs and their 5′ ends, but not their 3′ ends (Additional file 3: Figure S7). In this manner, peaks were called in both deep data sets (rep0, 23 °C and 37 °C) and corresponding genomic intervals were merged between data sets and between intervals if they overlapped more than 80%.

The resulting list of 5,600 genomic candidate intervals (sRNAs) was then subjected to differential expression (DE) analysis. We extracted count tables for these intervals from the two low-coverage replicates (rep1, rep2) and calculated DE with edgeR and DEseq [68, 69]. 5 intervals could not be tested due to too-low coverage in (one of) the replicates. Results were then filtered by adjusted *P-value* ≤ 0.05 (P-values were adjusted using Benjamini and Hochberg’s algorithm to control the false discovery rate). For further analyses we continued with only the edgeR data. We do, however, provide the adjusted DEseq P-values in the final result table for completeness.

Finally, all genomic intervals were manually assessed and curated by multiple members of our research group by visual inspection of the normalized coverage data in the IGV [70] genome browser. Besides obvious false positive calls and likely degradation products (Additional file 12: Figure S8), we also removed all peaks associated with annotated open reading frames that were 600 nucleotides or shorter from the list of sRNAs.

### Analysis of repetitive regions

The *B. burgdorferi* genome consists of a linear chromosome and up to 21 or more linear (lp) as well as circular (cp) plasmids. A considerable fraction of the plasmid DNA is highly repetitive, particularly some regions on the cp32s, lp56, lp21 and lp5 are nearly sequence-identical [71], which makes unambiguous read mapping in these regions difficult or impossible. This constituted two major problems for our analysis: first, we observed artificial coverage peaks around the borders to such repetitive regions that were wrongly classified as sRNA peaks by our method, resulting in false-positive calls. Secondly, we were aware that this would also lead to false negative (i.e., missed) peaks in repetitive regions (as we removed all multi-mapped reads from our datasets).

For this reason, we conducted an additional analysis that guided our manual curation of sRNA candidate regions. First, we remapped all multi-mapped reads (i.e., reads with mapping quality zero (MQ0), mapping accurately to more than one place in the genome) from our alignments using NextGenMap, this time configuring the mapper to additionally output up to 100 alignments that share the maximum alignment score per read (i.e., a read that was sequenced from a genomic region that has three perfect copies in the genome would be represented with three entries in the resulting BAM file). Then, we calculated the read coverage signals from these data as we did before, but this time each alignment contributed to the coverage at a particular genomic position only with a weight 1/X0, where X0 is the number of optimal alignments for this read in the dataset. In other words, a read that aligned to three different (repetitive) regions with the same maximum score would contribute with a “weight” of 1/3 to the each of them, thereby equally dividing its contribution among all possible (optimal) mapping locations in the genome. The resulting coverage tracks were converted to the BigWig [72] format and loaded into IGV along with our standard tracks for use in our manual curation. A peak was considered a false positive (due to MQ0 reads) if it had reads surrounding it in the MQ0 coverage map. For example, an intragenic peak was called in the *bbs02* gene on cp32-3, but manual inspection of the MQ0 reads shows good coverage of the proximal area around the peak, suggesting it is a false positive peak due to repetitive sequence surrounding it (Additional file 13: Figure S9). In addition, we likely missed sRNAs in these repetitive regions as well. For example, manual inspection of the MQ0 reads on cp32-1 revealed a putative antisense RNA opposite the *bbp17* gene. The uniquely mapped coverage maps do not have any reads mapped to this genomic region because it is repetitive with the other cp32 plasmids, but the MQ0 coverage map suggests at least one of these cp32 derived sequences are transcribed (Additional file 14: Figure S10).

### Northern blots

For Northern blot analysis 15 μg of DNase I (Roche) treated RNA was separated under denaturing conditions either by a 6–8% TBE-Urea (8 M) polyacrylamide gels in 1X TBE (for small transcripts) or a 1% formaldehyde/MOPS agarose gels in 1X MOPS (for larger transcripts). RNA was initially denatured in 2X RNA load dye (Fermentas) and heated to 65 °C for 15 min before loading on a gel. RNA was transferred to HybondXL membranes either by electroblotting at 12 V for 1 h in 0.5X TBE (polyacrylamide TBE-Urea gels) or capillary action (formaldehyde-agarose gels). The membranes were UV cross-linked and probed with DNA oligonucleotide probes (Additional file 15: Table S5). DNA oligonucleotide probes were end-labeled with gamma-32P [ATP] and T4 PNK (New England Biolabs) per the manufacturer’s protocol.

### Nucleotide context analysis

To validate the accuracy of our 5′ end peak-calling method on a genome-wide scale we analyzed the nucleotide composition around all identified 5′ ends. We calculated averaged nucleotide fractions in genomic windows centered at the most 5′ positions of identified sRNAs and plotted the results in Additional file 3: Figure S7. Overall, these data demonstrate an adenine and thymine (A and T) rich sequence at the −10 region correlating to the Pribnow box and an enrichment in thymine exactly 35 nucleotides upstream of the putative transcriptional start sites (TSS) across all novel sRNA categories described in this paper (as, IG, Intra, 5′ UTR), suggesting indeed these 5′ ends are accurate. Moreover, we didn’t detect these sequence elements associated with the annotated group of sRNAs, which are primarily tRNAs. tRNAs are processed from primary transcripts to their active and stable form and should not have a Pribnow box at the −10 region. The same plots were generated for all other sRNA subcategories used in this manuscript (as, IG, Intra, 5′ UTR), as well as for all sRNAs on the two different strands individually, and the respective signals were similar to the overall signal (the −35 T-peak being slightly less prominent for intra-RNAs). We have also generated respective plots for randomly chosen positions in the genome as a control, which showed, as expected, no major deviations from the genomic average (data can be found at http://www.cibiv.at/~niko/bbdb/). Together, these data provide additional *in silico* evidence for the ability of our method to accurately detect 5′ ends of small RNAs on a genome-wide scale.

## Additional files

Additional file 1: Figure S1. Peak calling proof of principle. The deep-sequencing results are displayed in a coverage map for two phenylalanine tRNAs. An overlay of the rep0 libraries sequenced deeply for sRNA peak calling (Peak) and the two biological replicates rep1, rep2 at both 23 °C and 37 °C are shown. The height at each position indicates the normalized number of reads that mapped to that base. The + strand coverage is shown in green. Note that the *y*-axis scale is different between the peak calling libraries (peak) and the biological replicates used for differential expression analyses (23 °C and 37 °C). The genomic context is illustrated below the coverage maps; black arrows indicate the annotated tRNA genes, the yellow boxes indicates the regions called as a small IG-RNAs by our peak finder (SR0159 and SR0160). (PDF 1169 kb)
Additional file 2: Table S1. Annotated list of sRNAs found in this study. The table contains information about the genomic locations of all manually curated sRNAs and data from temperature differential expression analysis (log-fold change and adjusted P-values for edgeR and DEseq). Previously annotated RNAs contain the respective name and/or function in the *Notes* column. Please note that *SsrA*, *ffs* and *RNaseP* were annotated based on their respective entries in the BSRD database where they were previously predicted by sequence similarity. (XLSX 234 kb)
Additional file 3: Figure S7. Nucleotide composition around the 5′ ends of detected small RNAs. The plots illustrate the averaged nucleotide fractions in genomic windows centered at the most 5′ position (position zero in the plots) of identified sRNAs. Panels A, C and E on the left show the data for all 1,005 sRNAs identified in this study. Panels B, D and F show the data for only the 37 previously annotated small RNAs, the majority (31) of which are tRNAs. Nucleotide distributions were calculated from the reference genome in a strand-specific manner. Panels A and B plot the sums of A + T and G + C fractions in a genomic window of 100 nucleotides up- and downstream of the putative 5′ ends. The horizontal grey lines were placed at the genomic averages for these measures (71.8% A + T, 28.2% G + C) and reveal that the genomic sequences upstream of the TSS are slightly more AT-rich, while the downstream (transcribed) regions are slightly more GC-rich compared to the genome-wide average (A). This effect is strongly pronounced for the annotated sRNAs (B). Panels C and D plot the contributions of the individual bases and panels E and F are zoomed-in versions of C and D (40 bp upstream to 5 bp downstream of the 5′ end). Dotted vertical grey lines highlight the genomic positions −10 and −35 relative to the 5′ end. Panels C and E show a Pribnow-box like element about 10 bases upstream of the 5′ end of the sRNAs, that is absent from the (mostly) tRNA-derived sequences (D and F). tRNAs are processed from primary transcripts to their active and stable form and should not have an Pribnow box at the −10 region. Furthermore, panels C and E reveal a strong T-peak at position −35 that may play a role in transcription initiation. Again, this feature is not detectable for the tRNA sequences. (PDF 19 kb)
Additional file 4: Table S2. Northern blot validation of sRNAs. The table summarizes the sRNAs that were confirmed via Northern blot analysis. The sRNAs location, category, sizes and associated genes are indicated. (XLSX 43 kb)
Additional file 5: Figure S2. Northern blot validation of asRNAs. Northern blot analyses of total RNA fractionated on a denaturing polyacrylamide gel, blotted to a nylon membrane, and hybridized with oligonucleotides specific for the asRNAs. The genomic context is illustrated above the Northern blots; the genes and RNAs are not drawn to scale. (PDF 982 kb)
Additional file 6: Figure S3. Northern blot validation of 5′ UTR sRNAs. Northern blot analyses of total RNA fractionated on a denaturing polyacrylamide gel, blotted to a nylon membrane, and hybridized with oligonucleotides specific for 5′ UTR RNAs. The genomic context is illustrated above the Northern blot; the genes and RNAs are not drawn to scale. (PDF 883 kb)
Additional file 7: Figure S4. Northern blot validation of intraRNAs. Northern blot analyses of total RNA fractionated on a denaturing polyacrylamide gel, blotted to a nylon membrane, and hybridized with oligonucleotides specific for intraRNAs. The genomic context is illustrated above the Northern blots; the genes and RNAs are not drawn to scale. (PDF 936 kb)
Additional file 8: Figure S5. Northern blot validation of IG-sRNAs. Northern blot analyses of total RNA fractionated on a denaturing polyacrylamide gel, blotted to a nylon membrane, and hybridized with oligonucleotides specific for IG-sRNAs. The genomic context is illustrated above the Northern blots; the genes and RNAs are not drawn to scale. (PDF 1096 kb)
Additional file 9: Figure S6 Northern blot validation of IG-sRNAs. Northern blot analyses of total RNA fractionated on a denaturing polyacrylamide gel, blotted to a nylon membrane, and hybridized with oligonucleotides specific for IG-sRNAs. The genomic context is illustrated above the Northern blots; the genes and RNAs are not drawn to scale. (PDF 1029 kb)
Additional file 10: Table S3. Genes with antisense and 5′ UTR sRNAs. The table contains information about all of the genes with antisense and 5′ UTR sRNAs associated with them. Gene ontology terms (GO terms) for biological processes (BP) are also given for each gene. The column titled, “Norgard FoldChange” contains the fold-change of that particular gene as reported by Revel et al. [52] via microarray after a temperature shift. (XLSX 221 kb)
Additional file 11: Table S4. Transcriptome data comparison. Comparison of genes reported as temperature-dependent in a DNA microarray [52] and asRNAs we identified opposite to them. (XLSX 11 kb)
Additional file 12: Figure S8. Manual curation of peaks. Intragenic RNA peaks called in genes *bb0311* and *bb0312* were manually curated based on the coverage patterns. The deep-sequencing results are displayed as described in the caption of Additional file 4: Figure S2. The - strand coverage is shown in blue. Note that the *y*-axis scale is different between the peak calling libraries (peak) and the biological replicates used for differential expression analyses (23 °C and 37 °C). The genomic context is illustrated below the coverage maps: black arrows indicate the annotated genes; the yellow box indicates the region called as a small intraRNA by our peak caller. The peaks appear to be broad and similar in height across the gene, suggesting they are degradation products of the mRNA, not stable sRNAs. (PDF 2229 kb)
Additional file 13: Figure S9. Manual curation of peaks based on MQ0 reads. The intragenic peak called in the *bbs02* gene was manually curated based on the MQ0 reads. The deep-sequencing results are displayed in a coverage maps for the *bbs02* gene. An overlay of the uniquely mapped rep0 libraries sequenced deeply for sRNA peak calling (Peak), the two biological replicates at both 23 °C and 37 °C (rep1, rep2) and the MQ0 reads from the deeply sequenced rep0 libraries are shown. The height at each position indicates the number of reads that mapped to that base. The + strand is shown in green. Note that the *y*-axis scale is different between the peak calling libraries (peak) and the biological replicates used for differential expression analyses (23 °C and 37 °C) and the MQ0 tracks. The genomic context is illustrated below the coverage maps: black arrows indicate the annotated genes; the yellow box indicates the region called as a small intra-RNA by our peak finder. (PDF 1120 kb)
Additional file 14: Figure S10. MQ0 reads reveal a cp32-derived asRNA. The MQ0 reads reveal an asRNA peak encoded opposite the *bbp17* gene. The deep-sequencing results are displayed in a coverage maps for the *bbp17* gene. An overlay of the uniquely mapped rep0 libraries sequenced deeply for sRNA peak calling (Peak), the two biological replicates (rep1, rep2) at both 23 °C and 37 °C and the MQ0 reads from the deeply sequenced rep0 libraries are shown. The height at each position indicates the number of reads that mapped to that base. The - strand is shown in blue. Note that the *y*-axis scale is different between the peak calling libraries (peak) and the biological replicates used for differential expression analyses (23 °C and 37 °C) and the MQ0 tracks. The genomic context is illustrated below the coverage maps: black arrows indicate the annotated genes. (PDF 904 kb)
Additional file 15: Table S5. Oligonucleotides used in this study. Sequences and names of all oligonucleotides used in this study. (DOCX 15 kb)

## Abbreviations

3′ UTR: 3′ untranslated region
5′ UTR: 5′ untranslated region
asRNA: Antisense small RNA
IG-RNA: Intergenic small RNA
intraRNA: Intragenic small RNA
sRNA: Small RNA

## References

[1] Mead PS (2015). Epidemiology of Lyme disease. Infect Dis Clin N Am.

[2] Lane RS, Piesman J, Burgdorfer W (1991). Lyme borreliosis: relation of its causative agent to its vectors and hosts in North America and Europe. Annu Rev Entomol.

[3] Piesman J, Schwan T, Samuels DS, Radolf JD (2010). Ecology of *borreliae* and their arthropod vectors. Borrelia: molecular biology, host interaction and pathogenesis.

[4] Radolf JD, Caimano MJ, Stevenson B, Hu LT (2012). Of ticks, mice and men: understanding the dual-host lifestyle of Lyme disease spirochaetes. Nat Rev Microbiol.

[5] Samuels DS (2011). Gene regulation in *Borrelia burgdorferi*. Annu Rev Microbiol.

[6] Schwan TG, Piesman J, Golde WT, Dolan MC, Rosa PA (1995). Induction of an outer surface protein on *Borrelia burgdorferi* during tick feeding. Proc Natl Acad Sci U S A.

[7] Alverson J, Bundle SF, Sohaskey CD, Lybecker MC, Samuels DS (2003). Transcriptional regulation of the *ospAB* and *ospC* promoters from *Borrelia burgdorferi*. Mol Microbiol.

[8] Yang X, Goldberg MS, Popova TG, Schoeler GB, Wikel SK, Hagman KE, Norgard MV (2000). Interdependence of environmental factors influencing reciprocal patterns of gene expression in virulent *Borrelia burgdorferi*. Mol Microbiol.

[9] Iyer R, Caimano MJ, Luthra A, Axline D, Corona A, Iacobas DA, Radolf JD, Schwartz I (2015). Stage-specific global alterations in the transcriptomes of Lyme disease spirochetes during tick feeding and following mammalian host adaptation. Mol Microbiol.

[10] Kazmierczak MJ, Wiedmann M, Boor KJ (2005). Alternative sigma factors and their roles in bacterial virulence. Microbiology and molecular biology reviews : MMBR.

[11] Hubner A, Yang X, Nolen DM, Popova TG, Cabello FC, Norgard MV (2001). Expression of *Borrelia burgdorferi* OspC and DbpA is controlled by a RpoN-RpoS regulatory pathway. Proc Natl Acad Sci U S A.

[12] Groshong AM, Blevins JS (2014). Insights into the biology of *Borrelia burgdorferi* gained through the application of molecular genetics. Adv Appl Microbiol.

[13] Caimano MJ, Drecktrah D, Kung F, Samuels DS (2016). Interaction of the Lyme disease spirochete with its tick vector. Cell Microbiol.

[14] Samuels DS, Radolf JD (2009). Who is the BosR around here anyway?. Mol Microbiol.

[15] Beisel CL, Storz G (2010). Base pairing small RNAs and their roles in global regulatory networks. FEMS Microbiol Rev.

[16] Waters LS, Storz G (2009). Regulatory RNAs in bacteria. Cell.

[17] Storz G, Vogel J, Wassarman KM (2011). Regulation by small RNAs in bacteria: expanding frontiers. Mol Cell.

[18] Caldelari I, Chao Y, Romby P, Vogel J (2013). RNA-mediated regulation in pathogenic bacteria. Cold Spring Harb Perspect Med.

[19] Vogel J, Luisi BF (2011). Hfq and its constellation of RNA. Nat Rev Microbiol.

[20] Georg J, Hess WR (2011). cis-antisense RNA, another level of gene regulation in bacteria. Microbiol Mol Biol Rev.

[21] Thomason MK, Storz G (2010). Bacterial antisense RNAs: how many are there, and what are they doing?. Annu Rev Genet.

[22] Lybecker M, Zimmermann B, Bilusic I, Tukhtubaeva N, Schroeder R (2014). The double-stranded transcriptome of *Escherichia coli*. Proc Natl Acad Sci U S A.

[23] Heidrich N, Brantl S (2007). Antisense RNA-mediated transcriptional attenuation in plasmid pIP501: the simultaneous interaction between two complementary loop pairs is required for efficient inhibition by the antisense RNA. Microbiology.

[24] Wagner EG, Simons RW (1994). Antisense RNA control in bacteria, phages, and plasmids. Annu Rev Microbiol.

[25] Brantl S, Wagner EG (1994). Antisense RNA-mediated transcriptional attenuation occurs faster than stable antisense/target RNA pairing: an in vitro study of plasmid pIP501. EMBO J.

[26] Breaker RR (2009). Riboswitches: from ancient gene-control systems to modern drug targets. Future Microbiol.

[27] Narberhaus F (2010). Translational control of bacterial heat shock and virulence genes by temperature-sensing mRNAs. RNA Biol.

[28] Ramesh A, Winkler WC (2010). Magnesium-sensing riboswitches in bacteria. RNA Biol.

[29] Marzi S, Romby P (2012). RNA mimicry, a decoy for regulatory proteins. Mol Microbiol.

[30] Romeo T, Vakulskas CA, Babitzke P (2013). Post-transcriptional regulation on a global scale: form and function of Csr/Rsm systems. Environ Microbiol.

[31] Bilusic I, Popitsch N, Rescheneder P, Schroeder R, Lybecker M (2014). Revisiting the coding potential of the *E. coli* genome through Hfq co-immunoprecipitation. RNA Biol.

[32] Novick RP (2003). Autoinduction and signal transduction in the regulation of *staphylococcal* virulence. Mol Microbiol.

[33] Novick RP, Geisinger E (2008). Quorum sensing in *staphylococci*. Annu Rev Genet.

[34] Gripenland J, Netterling S, Loh E, Tiensuu T, Toledo-Arana A, Johansson J (2010). RNAs: regulators of bacterial virulence. Nat Rev Microbiol.

[35] Papenfort K, Vogel J (2010). Regulatory RNA in bacterial pathogens. Cell Host Microbe.

[36] Lebreton A, Cossart P. RNA- and protein-mediated control of *Listeria monocytogenes* virulence gene expression. RNA Biol. 2016:1–11. Epub ahead of print. 10.1080/15476286.2016.1189069 PMC5449094 27217337

[37] Lybecker MC, Samuels DS (2007). Temperature-induced regulation of RpoS by a small RNA in *Borrelia burgdorferi*. Mol Microbiol.

[38] Ouyang Z, Zhou J, Norgard MV (2014). CsrA (BB0184) is not involved in activation of the RpoN-RpoS regulatory pathway in *Borrelia burgdorferi*. Infect Immun.

[39] Lybecker MC, Abel CA, Feig AL, Samuels DS (2010). Identification and function of the RNA chaperone Hfq in the Lyme disease spirochete *Borrelia burgdorferi*. Mol Microbiol.

[40] Sanjuan E, Esteve-Gassent MD, Maruskova M, Seshu J (2009). Overexpression of CsrA (BB0184) alters the morphology and antigen profiles of *Borrelia burgdorferi*. Infect Immun.

[41] Sze CW, Li C (2011). Inactivation of bb0184, which encodes carbon storage regulator A, represses the infectivity of *Borrelia burgdorferi*. Infect Immun.

[42] Thorvaldsdottir H, Robinson JT, Mesirov JP (2013). Integrative Genomics Viewer (IGV): high-performance genomics data visualization and exploration. Brief Bioinform.

[43] Kent WJ, Sugnet CW, Furey TS, Roskin KM, Pringle TH, Zahler AM, Haussler D (2002). The human genome browser at UCSC. Genome Res.

[44] Patton TG, Brandt KS, Nolder C, Clifton DR, Carroll JA, Gilmore RD (2013). *Borrelia burgdorferi bba66* gene inactivation results in attenuated mouse infection by tick transmission. Infect Immun.

[45] Caimano MJ, Kenedy MR, Kairu T, Desrosiers DC, Harman M, Dunham-Ems S, Akins DR, Pal U, Radolf JD (2011). The hybrid histidine kinase Hk1 is part of a two-component system that is essential for survival of *Borrelia burgdorferi* in feeding *Ixodes scapularis* ticks. Infect Immun.

[46] He M, Ouyang Z, Troxell B, Xu H, Moh A, Piesman J, Norgard MV, Gomelsky M, Yang XF (2011). Cyclic di-GMP is essential for the survival of the lyme disease spirochete in ticks. PLoS Pathog.

[47] Kostick JL, Szkotnicki LT, Rogers EA, Bocci P, Raffaelli N, Marconi RT (2011). The diguanylate cyclase, Rrp1, regulates critical steps in the enzootic cycle of the Lyme disease spirochetes. Mol Microbiol.

[48] Salman-Dilgimen A, Hardy PO, Dresser AR, Chaconas G (2011). HrpA, a DEAH-box RNA helicase, is involved in global gene regulation in the Lyme disease spirochete. PLoS One.

[49] Chaconas G, Stewart PE, Tilly K, Bono JL, Rosa P (2001). Telomere resolution in the Lyme disease spirochete. EMBO J.

[50] Jewett MW, Lawrence KA, Bestor A, Byram R, Gherardini F, Rosa PA (2009). GuaA and GuaB are essential for *Borrelia burgdorferi* survival in the tick-mouse infection cycle. J Bacteriol.

[51] Kobryn K, Chaconas G (2002). ResT, a telomere resolvase encoded by the Lyme disease spirochete. Mol Cell.

[52] Revel AT, Talaat AM, Norgard MV (2002). DNA microarray analysis of differential gene expression in *Borrelia burgdorferi*, the Lyme disease spirochete. Proc Natl Acad Sci U S A.

[53] Chao Y, Papenfort K, Reinhardt R, Sharma CM, Vogel J (2012). An atlas of Hfq-bound transcripts reveals 3′ UTRs as a genomic reservoir of regulatory small RNAs. EMBO J.

[54] Chao Y, Vogel J (2016). A 3′ UTR-Derived Small RNA Provides the Regulatory Noncoding Arm of the Inner Membrane Stress Response. Mol Cell.

[55] Lybecker M, Bilusic I, Raghavan R (2014). Pervasive transcription: detecting functional RNAs in bacteria. Transcription.

[56] Gossringer M, Hartmann RK (2012). 3′-UTRs as a source of regulatory RNAs in bacteria. EMBO J.

[57] Toledo-Arana A, Dussurget O, Nikitas G, Sesto N, Guet-Revillet H, Balestrino D, Loh E, Gripenland J, Tiensuu T, Vaitkevicius K (2009). The *Listeria* transcriptional landscape from saprophytism to virulence. Nature.

[58] Beaume M, Hernandez D, Francois P, Schrenzel J (2010). New approaches for functional genomic studies in staphylococci. Int J Med Microbiol.

[59] Sharma CM, Hoffmann S, Darfeuille F, Reignier J, Findeiss S, Sittka A, Chabas S, Reiche K, Hackermuller J, Reinhardt R (2010). The primary transcriptome of the major human pathogen *Helicobacter pylori*. Nature.

[60] Kortmann J, Narberhaus F (2012). Bacterial RNA thermometers: molecular zippers and switches. Nat Rev Microbiol.

[61] Narberhaus F, Waldminghaus T, Chowdhury S (2006). RNA thermometers. FEMS Microbiol Rev.

[62] Thomason MK, Bischler T, Eisenbart SK, Forstner KU, Zhang A, Herbig A, Nieselt K, Sharma CM, Storz G (2015). Global transcriptional start site mapping using differential RNA sequencing reveals novel antisense RNAs in *Escherichia coli*. J Bacteriol.

[63] Lluch-Senar M, Delgado J, Chen WH, Llorens-Rico V, O’Reilly FJ, Wodke JA, Unal EB, Yus E, Martinez S, Nichols RJ (2015). Defining a minimal cell: essentiality of small ORFs and ncRNAs in a genome-reduced bacterium. Mol Syst Biol.

[64] Mackowiak SD, Zauber H, Bielow C, Thiel D, Kutz K, Calviello L, Mastrobuoni G, Rajewsky N, Kempa S, Selbach M (2015). Extensive identification and analysis of conserved small ORFs in animals. Genome Biol.

[65] Drecktrah D, Lybecker M, Popitsch N, Rescheneder P, Hall LS, Samuels DS (2015). The *borrelia burgdorferi* RelA/SpoT homolog and stringent response regulate survival in the tick vector and global gene expression during starvation. PLoS Pathog.

[66] Martin M (2011). Cutadapt removes adapter sequences from high-throughput sequencing reads. EMBnetjournal.

[67] Quinlan AR, Hall IM (2010). BEDTools: a flexible suite of utilities for comparing genomic features. Bioinformatics.

[68] Robinson MD, McCarthy DJ, Smyth GK (2010). edgeR: a Bioconductor package for differential expression analysis of digital gene expression data. Bioinformatics.

[69] Anders S, Huber W (2010). Differential expression analysis for sequence count data. Genome Biol.

[70] Robinson JT, Thorvaldsdottir H, Winckler W, Guttman M, Lander ES, Getz G, Mesirov JP (2011). Integrative genomics viewer. Nat Biotechnol.

[71] Casjens S (2000). Borrelia genomes in the year 2000. J Mol Microbiol Biotechnol.

[72] Kent WJ, Zweig AS, Barber G, Hinrichs AS, Karolchik D (2010). BigWig and BigBed: enabling browsing of large distributed datasets. Bioinformatics.

