# Supplementary material for: Temperature-dependent sRNA transcriptome of the Lyme disease spirochete
Source: BMC Genomics. 2017 Jan 5;18:28. doi: 10.1186/s12864-016-3398-3 (PMC5216591; doi:10.1186/s12864-016-3398-3)
Supplement: Additional file 1: Figure S1. — Peak calling proof of principle. The deep-sequencing results are displayed in a coverage map for two phenylalanine tRNAs. An overlay of the rep0 libraries sequenced deeply for sRNA peak calling (Peak) and the two biological replicates rep1, rep2 at both 23 °C and 37 °C are shown. The height at each position indicates the normalized number of reads that mapped to that base. The + strand coverage is shown in green. Note that the y-axis scale is different between the peak calling libraries (peak) and the biological replicates used for differential expression analyses (23 °C and 37 °C). The genomic context is illustrated below the coverage maps; black arrows indicate the annotated tRNA genes, the yellow boxes indicates the regions called as a small IG-RNAs by our peak finder (SR0159 and SR0160). (PDF 1169 kb) [file 12864_2016_3398_MOESM1_ESM.pdf]

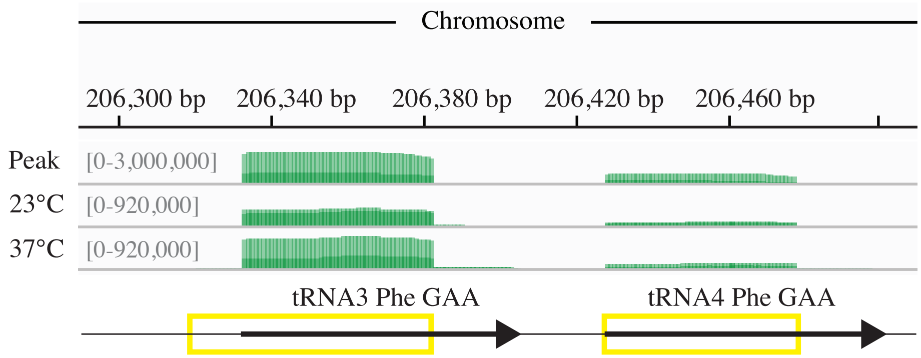

Figure S1. Peak calling proof of principle. The deep-sequencing results are displayed in a coverage map for two phenylalanine tRNAs. An overlay of the rep0 libraries sequenced deeply for sRNA peak calling (Peak) and the two biological replicates rep1, rep2 at both 23°C and 37°C are shown. The height at each position indicates the normalized number of reads that mapped to that base. The + strand coverage is shown in green. Note that the y-axis scale is different between the peak calling libraries (peak) and the biological replicates used for differential expression analyses (23°C and 37°C). The genomic context is illustrated below the coverage maps; black arrows indicate the annotated tRNA genes, the yellow boxes indicates the regions called as a small IG-RNAs by our peak finder (SR0159 and SR0160).
