# Supplementary figures and images for: Temperature-dependent sRNA transcriptome of the Lyme disease spirochete
Source: BMC Genomics. 2017 Jan 5;18:28. doi: 10.1186/s12864-016-3398-3 (PMC5216591; doi:10.1186/s12864-016-3398-3)

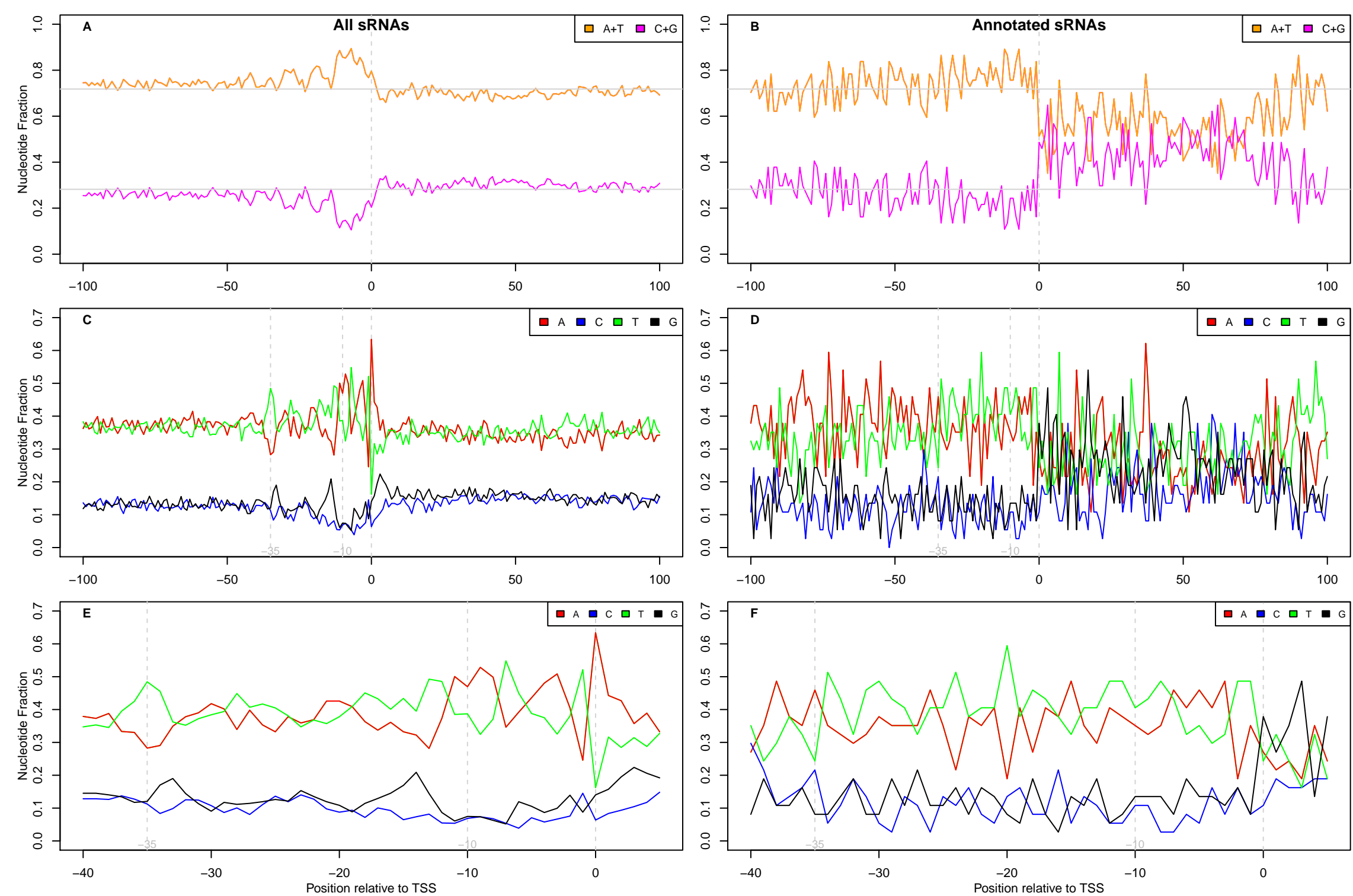

Supplement: Additional file 3: Figure S7. — Nucleotide composition around the 5′ ends of detected small RNAs. The plots illustrate the averaged nucleotide fractions in genomic windows centered at the most 5′ position (position zero in the plots) of identified sRNAs. Panels A, C and E on the left show the data for all 1,005 sRNAs identified in this study. Panels B, D and F show the data for only the 37 previously annotated small RNAs, the majority (31) of which are tRNAs. Nucleotide distributions were calculated from the reference genome in a strand-specific manner. Panels A and B plot the sums of A + T and G + C fractions in a genomic window of 100 nucleotides up- and downstream of the putative 5′ ends. The horizontal grey lines were placed at the genomic averages for these measures (71.8% A + T, 28.2% G + C) and reveal that the genomic sequences upstream of the TSS are slightly more AT-rich, while the downstream (transcribed) regions are slightly more GC-rich compared to the genome-wide average (A). This effect is strongly pronounced for the annotated sRNAs (B). Panels C and D plot the contributions of the individual bases and panels E and F are zoomed-in versions of C and D (40 bp upstream to 5 bp downstream of the 5′ end). Dotted vertical grey lines highlight the genomic positions −10 and −35 relative to the 5′ end. Panels C and E show a Pribnow-box like element about 10 bases upstream of the 5′ end of the sRNAs, that is absent from the (mostly) tRNA-derived sequences (D and F). tRNAs are processed from primary transcripts to their active and stable form and should not have an Pribnow box at the −10 region. Furthermore, panels C and E reveal a strong T-peak at position −35 that may play a role in transcription initiation. Again, this feature is not detectable for the tRNA sequences. (PDF 19 kb) [file 12864_2016_3398_MOESM3_ESM.pdf]
