# Supplementary material for: Temperature-dependent sRNA transcriptome of the Lyme disease spirochete
Source: BMC Genomics. 2017 Jan 5;18:28. doi: 10.1186/s12864-016-3398-3 (PMC5216591; doi:10.1186/s12864-016-3398-3)
Supplement: Additional file 8: Figure S5. — Northern blot validation of IG-sRNAs. Northern blot analyses of total RNA fractionated on a denaturing polyacrylamide gel, blotted to a nylon membrane, and hybridized with oligonucleotides specific for IG-sRNAs. The genomic context is illustrated above the Northern blots; the genes and RNAs are not drawn to scale. (PDF 1096 kb) [file 12864_2016_3398_MOESM8_ESM.pdf]

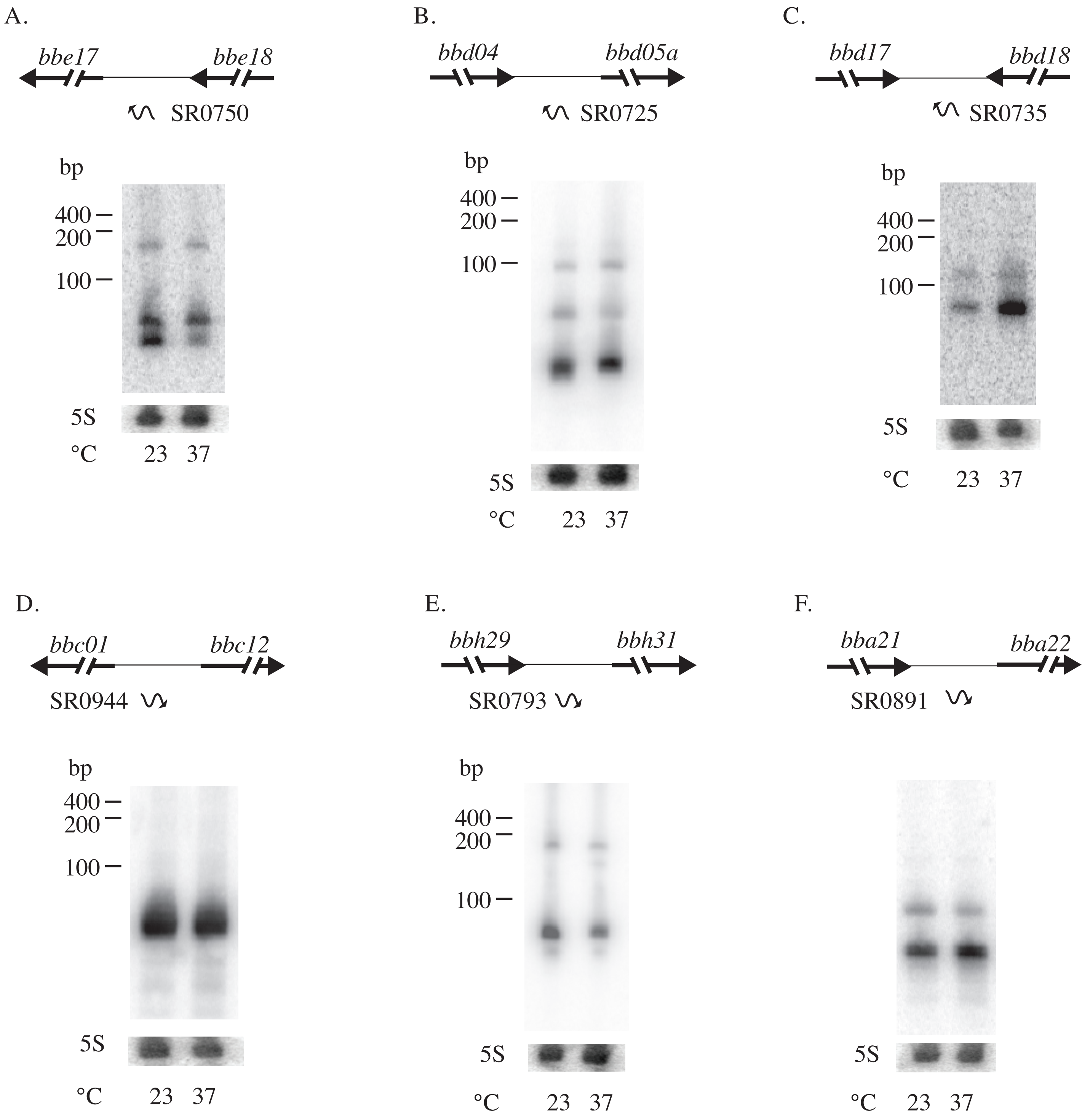

Figure S5. Northern blot validation of IG-sRNAs. Northern blot analyses of total RNA fractionated on a denaturing polyacrylamide gel, blotted to a nylon membrane, and hybridized with oligonucleotides specific for IG-sRNAs. The genomic context is illustrated above the Northern blots; the genes and RNAs are not drawn to scale.
