# Supplementary material for: Temperature-dependent sRNA transcriptome of the Lyme disease spirochete
Source: BMC Genomics. 2017 Jan 5;18:28. doi: 10.1186/s12864-016-3398-3 (PMC5216591; doi:10.1186/s12864-016-3398-3)
Supplement: Additional file 12: Figure S8. — Manual curation of peaks. Intragenic RNA peaks called in genes bb0311 and bb0312 were manually curated based on the coverage patterns. The deep-sequencing results are displayed as described in the caption of Additional file 4: Figure S2. The - strand coverage is shown in blue. Note that the y-axis scale is different between the peak calling libraries (peak) and the biological replicates used for differential expression analyses (23 °C and 37 °C). The genomic context is illustrated below the coverage maps: black arrows indicate the annotated genes; the yellow box indicates the region called as a small intraRNA by our peak caller. The peaks appear to be broad and similar in height across the gene, suggesting they are degradation products of the mRNA, not stable sRNAs. (PDF 2229 kb) [file 12864_2016_3398_MOESM12_ESM.pdf]

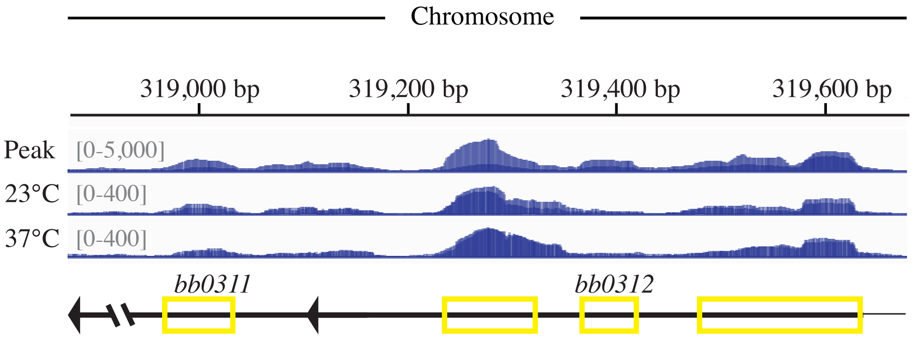

Figure S8. Manual curation of peaks. Intragenic RNA peaks called in genes *bb0311* and *bb0312* were manually curated based on the coverage patterns. The deep-sequencing results are displayed as described in the caption of Figure S2. The - strand coverage is shown in blue. Note that the y-axis scale is different between the peak calling libraries (peak) and the biological replicates used for differential expression analyses (23°C and 37°C). The genomic context is illustrated below the coverage maps: black arrows indicate the annotated genes; the yellow box indicates the region called as a small intraRNA by our peak caller. The peaks appear to be broad and similar in height across the gene, suggesting they are degradation products of the mRNA, not stable sRNAs.
