# Supplementary material for: Temperature-dependent sRNA transcriptome of the Lyme disease spirochete
Source: BMC Genomics. 2017 Jan 5;18:28. doi: 10.1186/s12864-016-3398-3 (PMC5216591; doi:10.1186/s12864-016-3398-3)
Supplement: Additional file 13: Figure S9. — Manual curation of peaks based on MQ0 reads. The intragenic peak called in the bbs02 gene was manually curated based on the MQ0 reads. The deep-sequencing results are displayed in a coverage maps for the bbs02 gene. An overlay of the uniquely mapped rep0 libraries sequenced deeply for sRNA peak calling (Peak), the two biological replicates at both 23 °C and 37 °C (rep1, rep2) and the MQ0 reads from the deeply sequenced rep0 libraries are shown. The height at each position indicates the number of reads that mapped to that base. The + strand is shown in green. Note that the y-axis scale is different between the peak calling libraries (peak) and the biological replicates used for differential expression analyses (23 °C and 37 °C) and the MQ0 tracks. The genomic context is illustrated below the coverage maps: black arrows indicate the annotated genes; the yellow box indicates the region called as a small intra-RNA by our peak finder. (PDF 1120 kb) [file 12864_2016_3398_MOESM13_ESM.pdf]

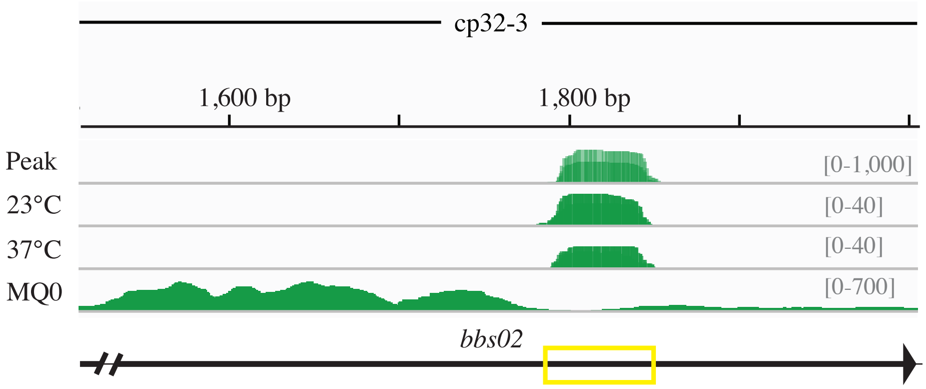

Figure S9. Manual curation of peaks based on MQ0 reads. The intragenic peak called in the *bbs02* gene was manually curated based on the MQ0 reads. The deep-sequencing results are displayed in a coverage maps for the bbs02 gene. An overlay of the uniquely mapped rep0 libraries sequenced deeply for sRNA peak calling (Peak), the two biological replicates at both 23°C and 37°C (rep1, rep2) and the MQ0 reads from the deeply sequenced rep0 libraries are shown. The height at each position indicates the number of reads that mapped to that base. The + strand is shown in green. Note that the y-axis scale is different between the peak calling libraries (peak) and the biological replicates used for differential expression analyses (23°C and 37°C) and the MQ0 tracks. The genomic context is illustrated below the coverage maps: black arrows indicate the annotated genes; the yellow box indicates the region called as a small intra-RNA by our peak finder.
