# Supplementary material for: Temperature-dependent sRNA transcriptome of the Lyme disease spirochete
Source: BMC Genomics. 2017 Jan 5;18:28. doi: 10.1186/s12864-016-3398-3 (PMC5216591; doi:10.1186/s12864-016-3398-3)
Supplement: Additional file 14: Figure S10. — MQ0 reads reveal a cp32-derived asRNA. The MQ0 reads reveal an asRNA peak encoded opposite the bbp17 gene. The deep-sequencing results are displayed in a coverage maps for the bbp17 gene. An overlay of the uniquely mapped rep0 libraries sequenced deeply for sRNA peak calling (Peak), the two biological replicates (rep1, rep2) at both 23 °C and 37 °C and the MQ0 reads from the deeply sequenced rep0 libraries are shown. The height at each position indicates the number of reads that mapped to that base. The - strand is shown in blue. Note that the y-axis scale is different between the peak calling libraries (peak) and the biological replicates used for differential expression analyses (23 °C and 37 °C) and the MQ0 tracks. The genomic context is illustrated below the coverage maps: black arrows indicate the annotated genes. (PDF 904 kb) [file 12864_2016_3398_MOESM14_ESM.pdf]

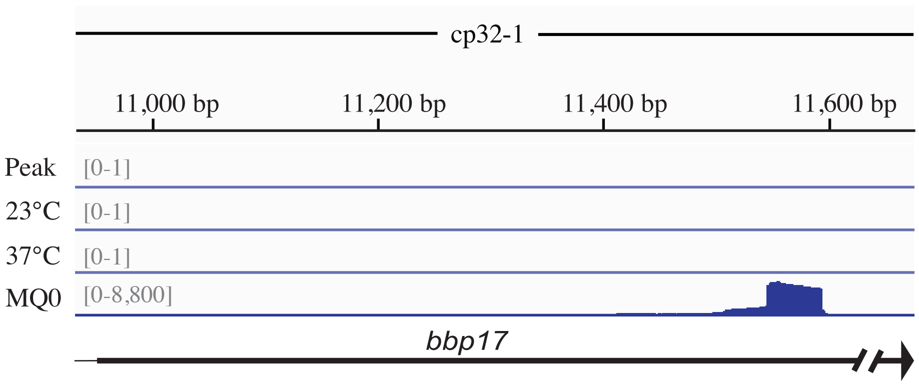

Figure S10. MQ0 reads reveal a cp32-derived asRNA. The MQ0 reads reveal an asRNA peak encoded opposite the *bbp17* gene. The deep-sequencing results are displayed in a coverage maps for the bbp17 gene. An overlay of the uniquely mapped rep0 libraries sequenced deeply for sRNA peak calling (Peak), the two biological replicates (rep1, rep2) at both 23°C and 37°C and the MQ0 reads from the deeply sequenced rep0 libraries are shown. The height at each position indicates the number of reads that mapped to that base. The - strand is shown in blue. Note that the y-axis scale is different between the peak calling libraries (peak) and the biological replicates used for differential expression analyses (23°C and 37°C) and the MQ0 tracks. The genomic context is illustrated below the coverage maps: black arrows indicate the annotated genes.
