# Supplementary material for: Temperature-dependent sRNA transcriptome of the Lyme disease spirochete
Source: BMC Genomics. 2017 Jan 5;18:28. doi: 10.1186/s12864-016-3398-3 (PMC5216591; doi:10.1186/s12864-016-3398-3)
Supplement: Additional file 15: Table S5. — Oligonucleotides used in this study. Sequences and names of all oligonucleotides used in this study. (DOCX 15 kb) [file 12864_2016_3398_MOESM15_ESM.docx]

| **Table S5**. Oligonucleotides for Northern Blots | |
| --- | --- |
| Name | Sequence (5′–3′) |
| SR104 | GGCGGAAAAACTGGTGTTAGTGTTTACTGGTGGAATTTAAATGCAT |
| SR315 | TATTGGCCTTAAAAAACGATAAAGTTGATTATATATATGGTGATTGCAA |
| SR491 | GTATGTTGTTGGGCAAGAAGATGCTAAAAAAGTCTTATCTGTGGCTGTT |
| SR688 | GGGTTAATTGTTTTAGTTATGATTATTTTTGCAAAGCCTATTATGTATTTTAT |
| SR953 | ATTGACTTTAAGGACAATTGGAACGTATCGCACAGTATAAAATTTAAT |
| SR750 | GACATCGACCAAAGTTAAGGATGCTTATAGTTAATAGCACCACTTACCAAG |
| SR891 | CCCTTTAAGGTAACAGGTCCTTATATTAAGAGCGACACATTGCGTGTCAGTCTGTACC |
| SR961 | CCTTGTGGTAGCCAACTTATTATTCTAATCAAACAACAAGAGCGAGACATTGCATCTCAATC |
| SR725 | CCACTTAAAGCCCTTTATCTGGTTATCATCCATTTGGAGCACATAATGCTTCCTAATT |
| SR921 | CGCTTTGATTGCGACGATAATTGTGGAAGGACAAACGACATCATCCAGCC |
| SR735 | GATCTTATTTGGAGAGGATTAATAGGGGTGTTGGGGACTATTGGTAGATCGTTTTCTACC |
| SR944 | GCCCTATGGATTTAAGAACTGATTATACTTACGTAGTAAAAATACTACAGA |
| SR956 | GAGTCAATAAGTTAAAATTTATGCCTACTTGATTGCAAATTTTTTATGCAATG |
| SR793 | GGGCAAATATTTTACTACTGAACACGGAGAGAGAAAAGCTCTCTAACC |
| SR888 | GGGTAGAGATTAATACAATCAAGTTGTGTTGGAAGTTCATTGTGGTTTTTTTTACT |
| SR891 | CCCTTTAAGGTAACAGGTCCTTATATTAAGAGCGACACATTGCGTGTCAGTCTGTACC |
| SR897 | GGCCTTAAGACCCTATTTGTAGTTTTAAAGAAGTTTTCAATGAATTGTTGATTTATAAC |
| SR899 | CAATGCTAGCAAAAGTAAGTAGAATATTTTGTAAGTTTTACTTATATTCTTT |
| SR902 | GCTTTTCTAAGCTTGCCGTTCAAAGCCTCTCACTCCTTTAAAAAAGAATTG |
| SR905 | GTTATTTCATATTGATTTAAACAGTCTACTGGCCTTGATGAAGTATGTAGCTATT |
| SR908 | CGTGAATGTTTGCAAATTGAGCTCTTGAATTTTTTGATCTTAAGGTGTCA |
| SR915 | CTTTTTTTAAGCTTACAGCCCAGATCCAAATAAATCTAAATAAATTCAATC |
